# Bacterial and Fungal Endophytic Microbiomes of *Salicornia europaea*

**DOI:** 10.1128/AEM.00305-19

**Published:** 2019-06-17

**Authors:** Bliss Ursula Furtado, Marcin Gołębiewski, Monika Skorupa, Piotr Hulisz, Katarzyna Hrynkiewicz

**Affiliations:** a Department of Microbiology, Faculty of Biology and Environmental Protection, Nicolaus Copernicus University, Toruń, Poland; b Department of Plant Physiology and Biotechnology, Nicolaus Copernicus University, Toruń, Poland; c Interdisciplinary Center for Modern Technologies, Nicolaus Copernicus University, Toruń, Poland; d Department of Soil Science and Landscape Management, Faculty of Earth Sciences, Nicolaus Copernicus University, Toruń, Poland; University of Toronto

**Keywords:** 16S rRNA and ITS amplicon sequencing, endophyte, halophyte, microbial community structure, soil salinity

## Abstract

We examined *Salicornia europaea*, a nonmycorrhizal halophyte associated with specific and unique endophytic bacteria and fungi. The microbial community structure was analyzed at two sites differing in salinization history (anthropogenic and naturally saline site), in contrasting seasons (spring and fall) and in two plant organs (shoots and roots) via 16S rRNA and internal transcribed spacer amplicon sequencing. We observed distinct communities at the two sites, and in shoots and roots, while the season was of no importance. The bacterial community was less diverse in shoot libraries than in roots, regardless of the site and season, whereas no significant differences were observed for the fungal community. *Proteobacteria* and *Bacteroidetes* dominated bacterial assemblages, and *Ascomycetes* were the most frequent fungi. A root core microbiome operational taxonomic unit belonging to the genus *Marinimicrobium* was identified. We detected a significant influence of the *Salicornia* bacterial community on the fungal one by means of cocorrespondence analysis. In addition, pathways and potential functions of the bacterial community in Salicornia europaea were inferred and discussed. We can conclude that bacterial and fungal microbiomes of *S. europaea* are determined by the origin of salinity at the sites. Bacterial communities seemed to influence fungal ones, but not the other way around, which takes us closer to understanding of interactions between the two microbial groups. In addition, the plant organs of the halophyte filter the microbial community composition.

**IMPORTANCE** Endophytes are particularly fascinating because of their multifaceted lifestyle, i.e., they may exist as either free-living soil microbes or saprobic ones or pathogens. Endophytic communities of halophytes may be different than those in other plants because salinity acts as an environmental filter. At the same time, they may contribute to the host’s adaptation to adverse environmental conditions, which may be of importance in agriculture.

## Abstract (precis)

Endophytes are particularly fascinating because of their multifaceted lifestyle, i.e., they may exist as either free-living soil microbes or saprobic ones or pathogens. Endophytic communities of halophytes may be different than those in other plants because salinity acts as an environmental filter. At the same time, they may contribute to the host’s adaptation to adverse environmental conditions, which may be of importance in agriculture.

## INTRODUCTION

Halophytes are salt-tolerant plants that usually grow and survive in environments with salt concentrations as high as 1 M (1). They may play a role in the remediation of salt-affected (2) and heavy-metal-contaminated (3) environments and are widely utilized as an option to tackle the worldwide problem of decreasing area of cultivated lands (4).

The salt accumulating obligate halophyte Salicornia europaea L. (*Amaranthaceae*) is commonly found in coastal and inland salt marshes (5) and is cultivated in some countries for its culinary values (6). Its geographical distribution spans four continents: North America, Asia, Africa, and Europe (7). Research on halophyte plants is of particular interest today due to their diverse strategies to survive in such harsh environments. Halophytes developed several primary and secondary mechanisms to cope with salinity (8, 9). The primary mechanisms include osmotic adjustment, the exclusion of Na^+^ from the cell or plant tissue, or the isolation of Na^+^ in the vacuole (8). The secondary mechanisms consist of associated microorganisms that ameliorate plant growth and fitness, particularly under stress conditions (9). These microorganisms include arbuscular mycorrhizal fungi (AMF) that are known to protect plants under stress conditions by providing nutrients and maintain a better ion balance (10). However, some halophytes belong to typical nonmycorrhizal plants, e.g., Armeria maritima, Limonium vulgare, Juncus gerardii, and Triglochin maritima (11). Only a few publications revealed very low (<1%) colonization of these halophytes by AMF (11–13). However, even these results can be dubious due to the possibility of spores coming from adjacent mycorrhizal plants being nonspecifically attached to otherwise-noncolonized roots. Given the uncertainty of AMF associations with the roots of *S. europaea*, specific and unique endophytes inhabiting this halophyte could compensate for missing symbiotic protection against, e.g., abiotic stress or nutrient deficiency (14, 15). Endophytes are a group of microorganisms (include bacteria, fungi, and actinomycetes) that colonize the internal tissues of healthy living plant hosts without causing harm or symptoms of disease (16). They coevolve in their plant host to adapt themselves in the plant environment. Some endophytes produce phytochemicals, bioactive secondary compounds to increase plant growth and development, as well as improve plant host fitness during abiotic and biotic stress conditions (reviewed in references 9 and 17).

In this study, we assessed two sites differing in salinization history. One of them is a naturally saline site where brines emerge from Zechstein salt deposits, giving the plants an opportunity to coevolve with halophilic microorganisms for a long time (18). At the other site, salinity originated from soda factory wastewater ponds and has lasted for only 50 years (19). The two sites are only 40 km from one another, and the soil physicochemistry is similar, apart from the salinity origin. This system gives a unique opportunity to observe differences due to the various times of plant host-microbiome coevolution. Therefore, we first hypothesized that the *Salicornia* endophytic community composition at the two sites would be different, but a set of common microorganisms (a core microbiome) can be delineable.

Plants can provide niches for microbes under unfavorable environmental conditions and assist the microbes in reducing environmental stress. There is clear evidence on the role of endophytes from stressed environments and the benefits their host receives in this association (20). This is why our second hypothesis is that *S. europaea*, belonging to a small group of nonmycorrhizal plants, would bear a unique assemblage of bacterial and fungal endophytes being halotolerant that perform various ecological roles in protecting the host plant under saline conditions.

Although metagenomic studies on bacterial or fungal endophytes of crop species have been performed (21–23), few reports have discussed the composition of endophytic community in species with the ability to accumulate salts (24, 25), and no studies have simultaneously analyzed the two communities. Thus, our third and last hypothesis evaluated whether there was an influence of the bacterial endophytic community on the fungal one and vice versa.

To address all three hypotheses, we used a culture-independent (Illumina sequencing of 16S rRNA and internal transcribed spacer [ITS] amplicons) approach to simultaneously analyze the bacterial and fungal endophytic community in *S. europaea*. The results obtained can contribute to agriculture technologies, leading to enhanced production of nonmycorrhizal crops in saline soils.

## RESULTS

### Soil physicochemical analysis.

Table 1 lists the soil characteristics of the two sampling sites, site 1 (S1) and site 2 (S2), during two seasons: fall and spring. The pH was neutral and nearly the same at both sites during the two seasons. In addition, carbonates (%) and total organic carbon (TOC; %) were similar at both sites. The S1 soil had significantly higher Na^+^, Cl^–^, CaCO_3_, Mg^2+^, and EC_e_ values (two-way analysis of variance [ANOVA], *P* < 0.05) and significantly lower levels of Ca^2+^ (two-way ANOVA, *P* < 0.05) than S2 soil. There were no significant differences in other physicochemical properties between the S1 and S2 soils.

**TABLE 1:** Chemical parameters of soils from the two sampling areas: site 1 and site 2 in fall 2015 and spring 2016

Parameter	Mean ± SEM (*n* = 9)^*a*^			
	Site 1		Site 2	
	Fall 2015	Spring 2016	Fall 2015	Spring 2016
EC_e_	100.5 ± 27.6^B^	51.1 ± 12.7^A^	76.0 ± 19.5^C^	59.7 ± 12.2^A^
pH_e_	6.8 ± 0.1^A^	7.8 ± 0.1^B^	6.9 ± 0.1^A^	7.3 ± 0.1^B^
Na^+^ (g ⋅ dm^−3^)	21.5 ± 7.9^A^	9.2 ± 2.4^C^	11.8 ± 7.4^B^	7.4 ± 2.1^CB^
Cl^−^ (g ⋅ dm^−3^)	65.3 ± 21.6^A^	30.8 ± 5.9^C^	44.1 ± 13.4^B^	34.2 ± 5.6^C^
Ca^2+^ (g ⋅ dm^−3^)	4.2 ± 3.5^B^	0.9 ± 0.2^C^	8.1 ± 3.3^A^	7.6 ± 1.5^A^
K^+^ (g ⋅ dm^−3^)	0.4 ± 0.2^A^	0.2 ± 0.0^C^	0.2 ± 0.2^B^	0.2 ± 0.1^C^
Mg^2+^ (g ⋅ dm^−3^)	0.5 ± 0.2^A^	0.2 ± 0.1^B^	0.3 ± 0.2^B^	0.1 ± 0.0^C^
SO_4_^2−^ (g ⋅ dm^−3^)	0.3 ± 0.085^A^	0.8 ± 0.2^B^	0.1 ± 0.1^A^	0.6 ± 0.3^C^
HCO_3_^−^ (g ⋅ dm^−3^)	0.1 ± 0.0^A^	0.2 ± 0.1^A^	0.1 ± 0.0^A^	0.1 ± 0.0^A^
SP (%)	94.5 ± 14.1^A^	83.0 ± 9.3^B^	89.4 ± 10.5^B^	133.1 ± 48.6^C^
TOC (%)	5.9 ± 2.5^A^	4.8 ± 3.1^A^	7.5 ± 5.5^B^	3.3 ± 2.4^A^
CaCO_3_ (%)	39.4 ± 7.1^A^	33.9 ± 9.4^A^	28.4 ± 10.5^B^	23.1 ± 2.0^C^

a Values labeled with the same superscript capital letters are not significantly different (*P* ≤ 0.05). Abbreviations: ECe, electrical conductivity of the saturated extract; TOC, total organic carbon; SP, saturation percentage.

### Sequencing results.

A total of 1,841,573 high-quality sequences were retrieved after denoising, merging, and chimera checking. The number of reads per sample ranged from 2,031 to 36,430. After separating bacterial and fungal reads, 236,835 bacterial sequences (2 to 13,125 per sample) and 260,575 fungal sequences (6 to 14,969 per sample) were left. We eliminated 3 bacterial and 15 fungal libraries due to not reaching the required number of sequences (500 for bacterial and 300 for fungal libraries).

### Alpha- and beta-diversity in *S. europaea* endophytic communities.

**FIG 1:**
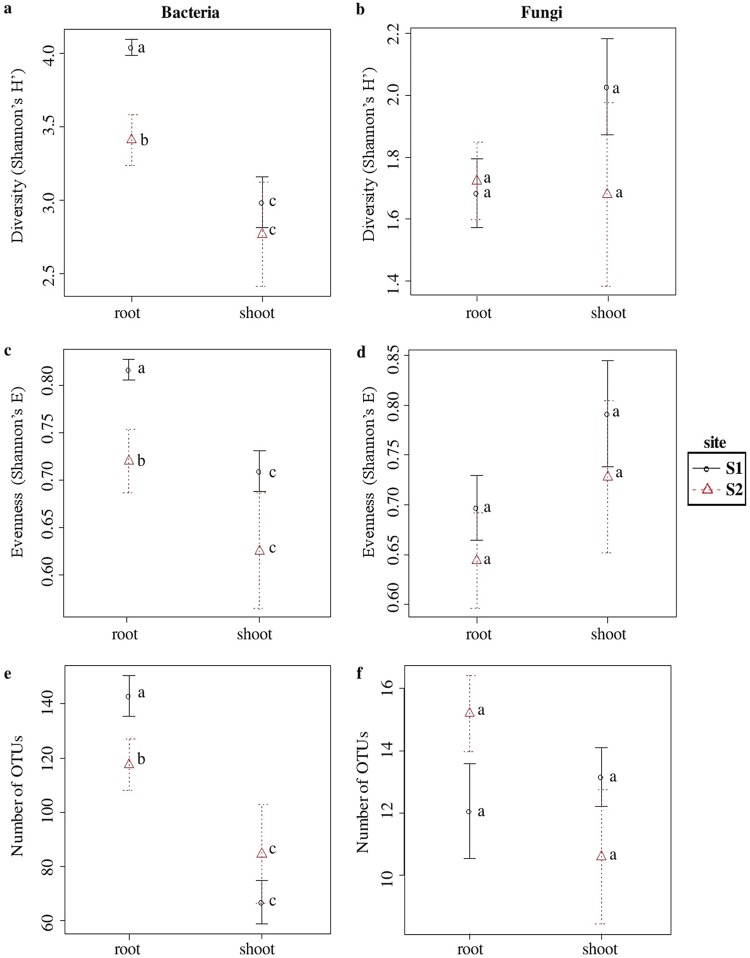
Species richness, diversity, and evenness in different test sites and plant organs for OTUs constructed at 0.03 dissimilarity for bacterial and fungal sequences. (a and b) Shannon’s H′; (c and d) Shannon’s E; (e and f) observed number of OTUs. Robust ANOVA test with the Tukey’s *post hoc* analysis was used to assess the significance of differences between test sites and plant organs. Variants labeled with the same letters are not significantly different (*P* ≤ 0.05).

No differences due to the season were observed in any of measured alpha-diversity indices both for bacterial and fungal communities. The bacterial Shannon’s diversity (H′) and evenness (E), as well as species richness, were lower in libraries derived from *S. europaea* shoots than in those from roots, regardless of site (Fig. 1a, c, and e). Differences were significant (ANOVA, *P* < 0.05) in the case of the S1 site, while for S2 they were not significant (ANOVA, *P* > 0.05). Unlike bacterial communities, no significant differences due to organs and sites were found for fungi. However, a tendency of greater diversity in shoots was visible for S2 libraries (Fig. 1b, d, and f).

**FIG 2:**
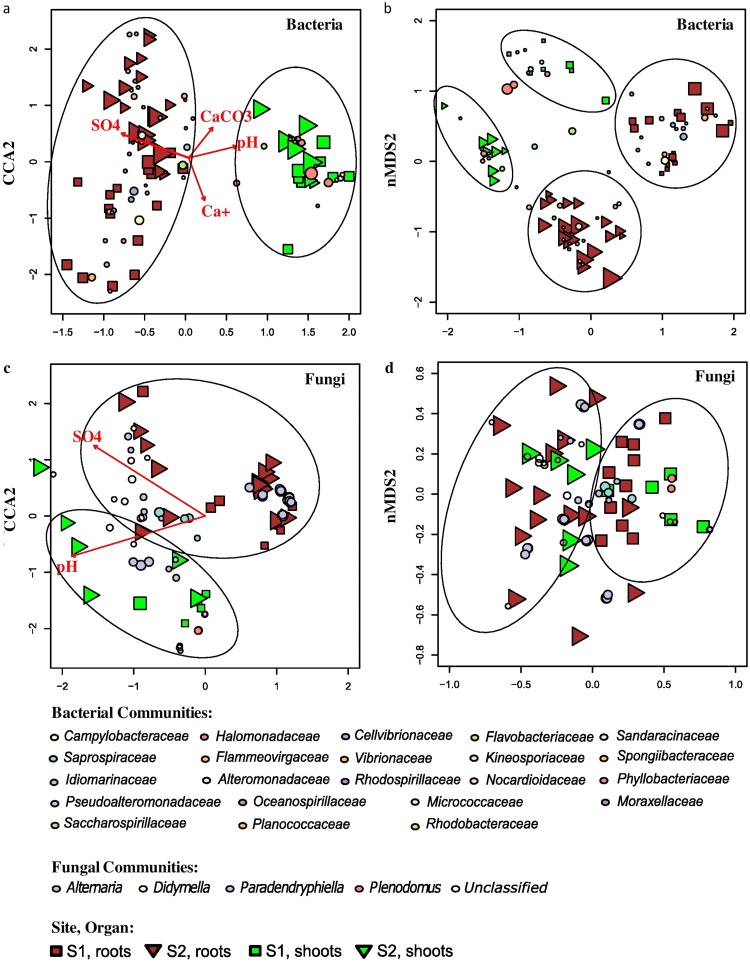
Analysis of log-transformed and Wisconsin double-standardized Bray-Curtis dissimilarity matrix for endophytic bacterial and fungal communities associated with *S. europaea*, respectively. (a and c) CCA (canonical correspondence analysis); (b and d) NMDS (nonmetric multidimensional scaling analysis). Circles represent OTUs; their fill color denotes consensus taxonomy at the family level (bacteria) and genus level (fungi). The size reflects abundance. Squares, S1 samples; triangles, S2 samples; green, shoots; brown, roots. Arrows in the CCA graphs denote soil parameters that were significantly associated with the community structure.

Beta-diversity analysis revealed that bacterial communities clustered according to site and organ in nonmetric multidimensional scaling (NMDS) (Fig. 2b), the grouping was significant (permutational analysis of variance [PERMANOVA], *P* < 0.01), and the differences in variance were not significant (PERMDISP, *P* > 0.01). Notably, the samples did not cluster according to the season. The distance between bacterial communities in the ordination plot for the roots was larger compared to the shoots. The canonical correspondence analysis (CCA) showed that Ca^+^, SO_4_^2+^_,_ CaCO_3_, and pH were the significant physicochemical variables influencing the community structure (Fig. 2a; *P* < 0.01). These variables explained 22.65% of the variance in the communities. The variations observed in the endophytic bacterial communities of the roots in S1 and S2 were distinctly high compared to the community present in the shoots.

Unlike bacterial ones, fungal communities clustered according to the site only (Fig. 2d), and this grouping was significant (PERMANOVA, *P* < 0.01), while variance was homogenous (PERMDISP, *P* > 0.01). pH and SO_4_^2+^ were the only physicochemical variables that significantly influenced the communities (Fig. 2c; *P* < 0.01). These variables explained 7.07% of the variance in the communities. In CCA, the fungal community variations were explained only by plant organs and not by the two different sites, confirming the results of PERMANOVA.

### Core microbiome.

A single bacterial OTU12 (a member of the *Marinimicrobium* genus) was found to exceed the abundance threshold in all root samples and might comprise *Salicornia* roots core microbiome. However, there were neither bacterial nor fungal operational taxonomic units (OTUs) with abundance greater than 0.5% in all samples.

### Bacterial and fungal community composition.

**FIG 3:**
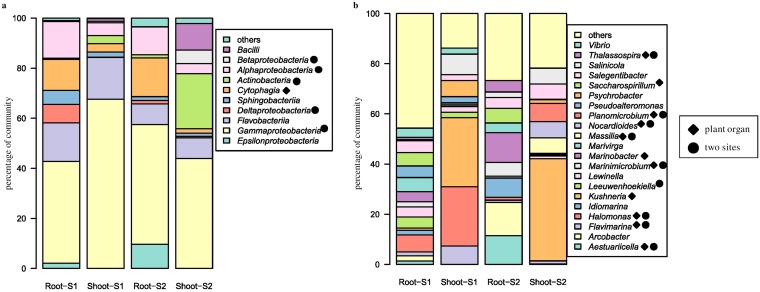
Endophytic bacterial community structure at class (a) and genus (b) levels among the two test sites and plant organs.

**FIG 4:**
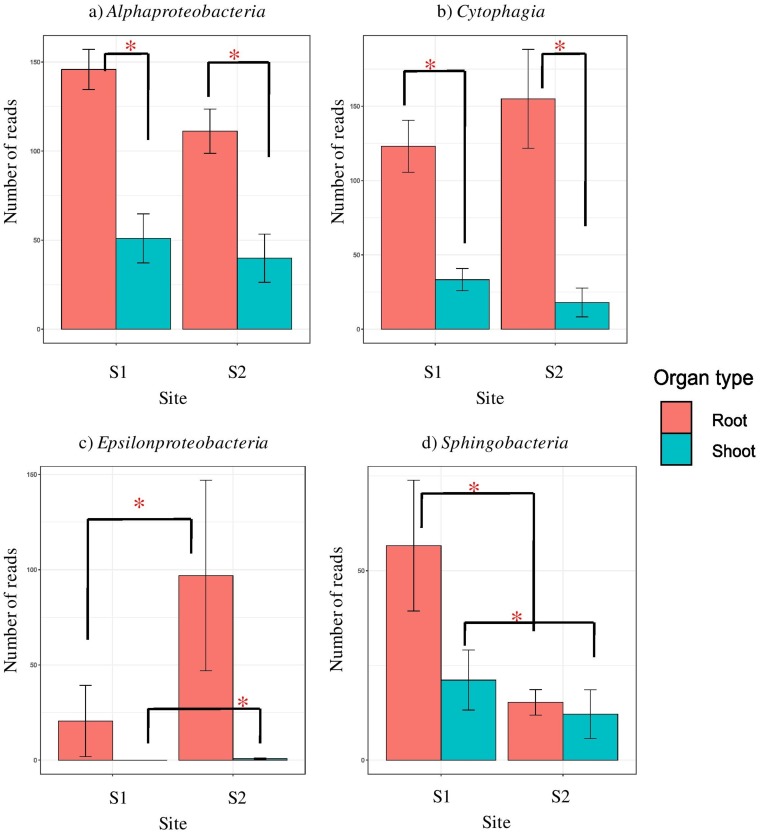
Significantly represented bacterial classes. (a) *Alphaproteobacteria*; (b) *Cytophagia*; (c) *Epsilonproteobacteria*; (d) *Sphingobacteriia*. Whiskers denote the standard errors of the mean. Significant differences (*P* < 0.05), assessed using robust ANOVA and Tukey’s HSD test, are indicated by asterisks.

**FIG 5:**
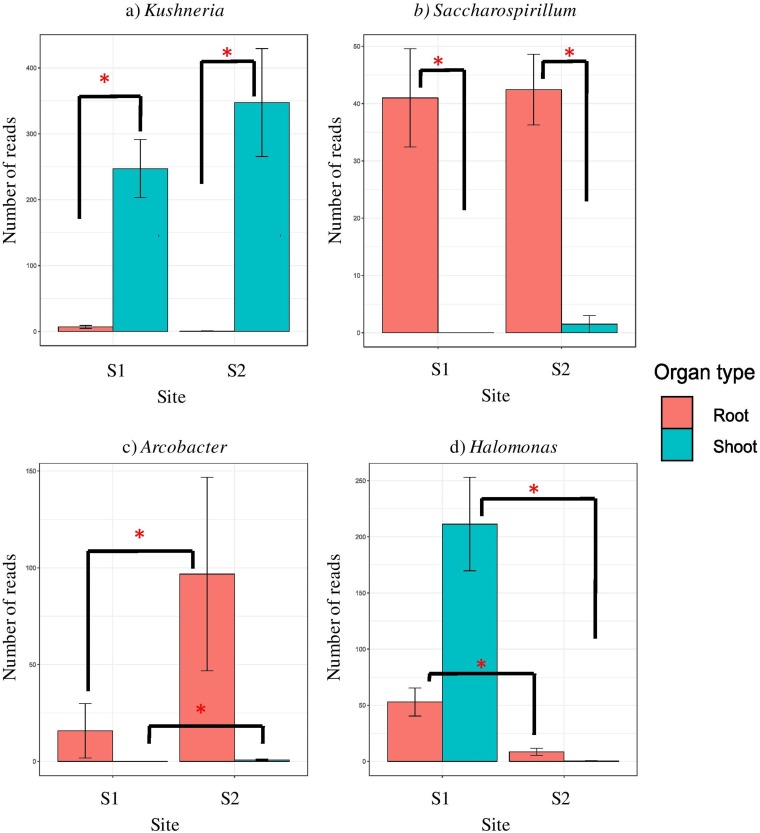
Significantly represented bacterial genera. (a) *Kushneria*; (b) *Saccharospirillum*; (c) *Arcobacter*; (d) *Halomonas*. Whiskers denote standard errors of the mean. Significant differences (*P* < 0.05), assessed using robust ANOVA and Tukey’s HSD test, are indicated by asterisks.

Bacterial libraries were dominated by *Proteobacteria*- and *Bacteroidetes*-derived reads (see Fig. S3A in the supplemental material). At the level of class, all the libraries, regardless of site and organ, were composed mainly of *Gammaproteobacteria* and *Flavobacteriia*, with minor amounts of *Alphaproteobacteria*, and *Cytophagia* (Fig. 3). The latter two classes were more frequent in roots than in shoots (Fig. 4a and b). Certain classes were specific for some sample types. *Sphingobacteriia* were found only in the S1 libraries (Fig. 4d), while *Epsilonproteobacteria* were characteristic for S2 (Fig. 4c). Deltaproteobacteria were specific for roots at S1 and *Betaproteobacteria* as well as *Bacilli* were characteristic for shoots at S2 (Fig. 3a). At the level of family, *Halomonadaceae* were much more abundant in shoots, whereas *Alteromonadaceae*, *Cellvibrionaceae*, *Flammeovirgaceae*, *Rhodobacteraceae*, and *Saccharospirillaceae* were characteristic for roots. *Vibrionaceae* and *Sandaracinaceae* were found exclusively at S1 (Fig. S3B). Thirty-one genera were found in the libraries, and shoot libraries were more similar between sites than root ones. *Kushneria* was characteristic for and abundant in shoots (Fig. 5a), while *Saccharospirillum* was a hallmark of roots (Fig. 5b). *Halomonas*, *Levinella*, *Vibrio*, *Pseudoalteromonas*, and *Leuweenhoekiella* were found exclusively at S1 (Fig. 5d). *Arcobacter* was present in minor quantities, but it was more abundant at S2 (Fig. 5c).

**FIG 6:**
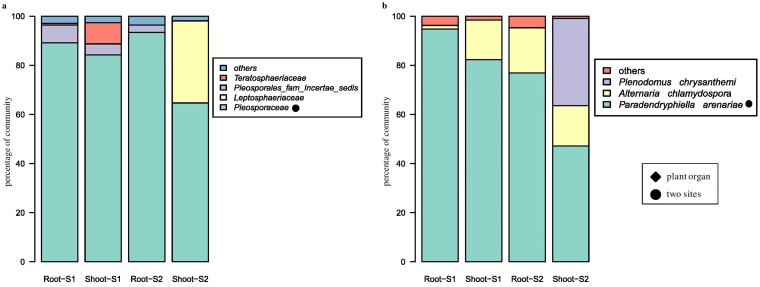
Endophytic fungal community structure at family (a) and species (b) levels among the two test sites and plant organs.

**FIG 7:**
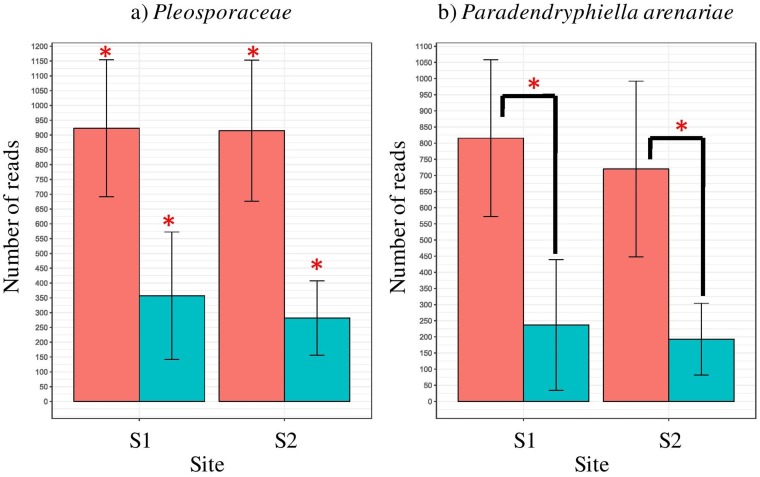
Significantly represented fungal taxa. (a) *Pleosporaceae*; (b) *Paradendryphiella arenariae*. Whiskers denote standard errors of the mean. Significant differences (*P* < 0.05), assessed using robust ANOVA and Tukey’s HSD test, are indicated by asterisks.

At high phylogenetic levels, no differences between sites and organs were visible in fungal communities: libraries were composed of over 95% *Ascomycota* reads derived from *Dothideomycetes* class (Fig. S4A and B). *Pleosporaceae* was the dominating family in all sample types; however, it was more abundant in roots than in shoots. *Pleosporales* fam. *incertae sedis* was found in all sample types but shoots at S2, albeit in small amounts (Fig. 6a). *Leptosphaeriaceae*, *Teratosphaeriaceae*, and *Didymosphaeriaceae* were found exclusively in shoots, the first of them at S2, while the remaining two at S1. Over 80% of fungal reads were classified down to the species level (Fig. 6b). *Paradendryphiella arenariae* was the only species present in all sample types, but more frequent in roots (Fig. 7b). An unclassified member of the *Pleosporaceae* made up a significant portion of the community in shoots but was also found in roots at S1 (Fig. 7a), whereas *Alternaria chlamydospora* was found in shoots and roots at S2.

### Bacterial metabolic pathways differentially represented in roots and shoots.

**FIG 8:**
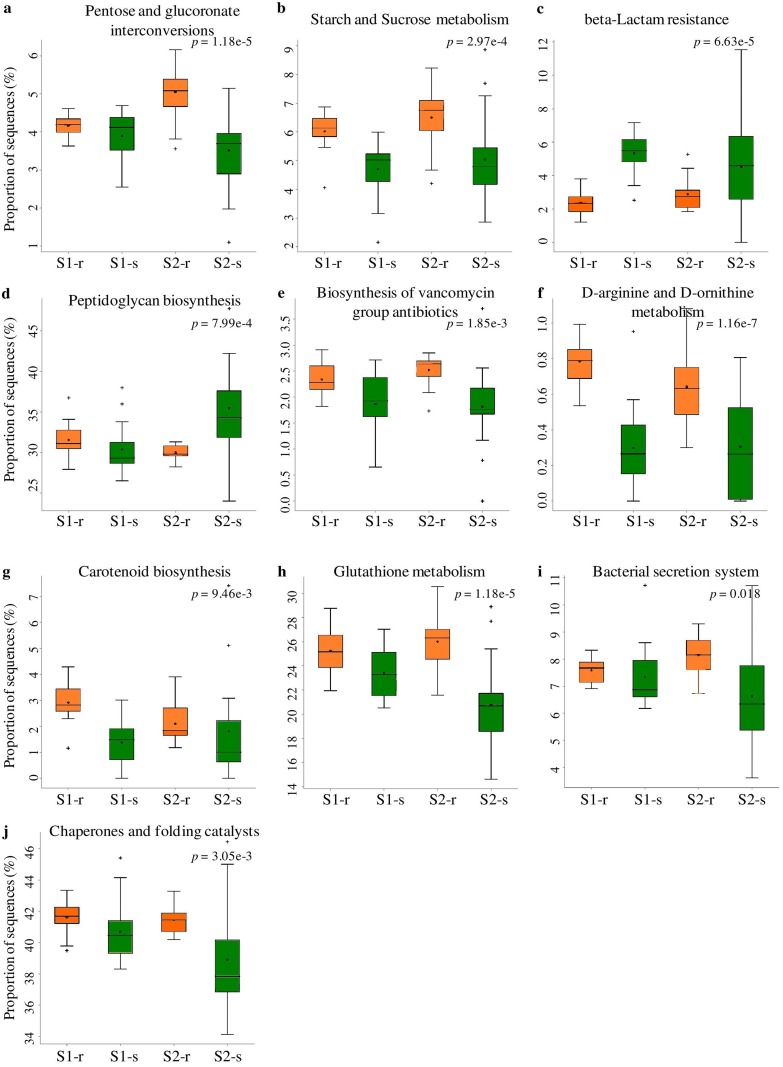
PICRUSt analysis of bacterial sequences. Box-and-whisker plots for most significantly different KEGG Orthology (KO) categories. Error bars represent standard errors of the mean of each given category abundance in different sample types. For sites S1 and S2, “-r” indicates “root” and “-s” indicates “shoot.”

Possible bacterial metagenomes were imputed using PICRUSt with GreenGenes as the underlying database. The nearest sequenced taxon index (NSTI) values were around 0.05 in the shoot and 0.09 in root samples, regardless of site and season. This difference was statistically significant (ANOVA, *P* < 0.01, *F* = 23.071). Ten level-3 pathways were differently represented in simulated metagenomes (Fig. 8): two of them belonged to carbohydrate metabolism (pentose-glucuronate interconversions, starch and sucrose metabolism) and three to antibiotic resistance and biosynthesis (beta-lactam resistance, peptidoglycan biosynthesis, and vancomycin biosynthesis), and the remaining ones came from host interactions (bacterial secretion system), amino acid metabolism (arginine and ornithine metabolism), protein biosynthesis (chaperones and folding catalysts), and regulation of redox potential (glutathione metabolism), as well as secondary metabolite biosynthesis (carotenoid biosynthesis). The majority of the pathways were overrepresented in root sample metagenomes; only beta-lactam resistance and peptidoglycan biosynthesis were overrepresented in shoots.

### Interactions between endophytic bacterial and fungal communities.

**FIG 9:**
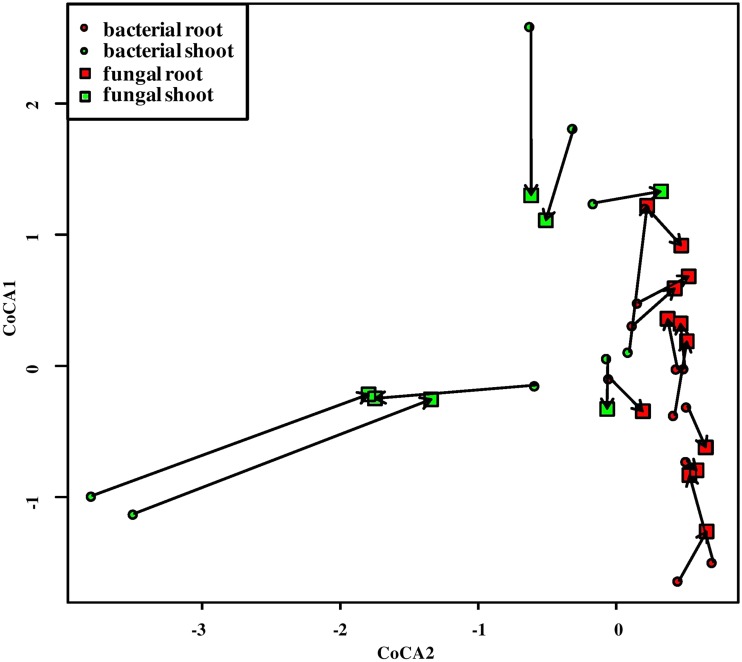
CoCA of bacterial versus fungal communities’ plot of sites. Bacterial sites are displayed as circles, fungal ones as squares. The circle color denotes organ: green, shoots; and red, roots. Respective bacterial and fungal communities are connected with arrows. Arrow length conveys the strength of community association: the longer the arrow, the weaker the association.

Cocorrespondence analysis (CoCA) indicated that there was a significant influence of the bacterial community on the fungal one and vice versa. In other words, the fungal community might be predicted based on the bacterial one. Leave-one-out cross-validation showed that two first axes were sufficient, and permutational testing demonstrated that only the first one was significant (permutest, *P* < 0.01). Distances between respective bacterial and fungal communities were significantly greater for shoot samples (1.21) than for roots (0.40) (ANOVA, *P* < 0.01, *F* = 13.73; Fig. 9), indicating that the influence was greater in the latter ones. Bacterial community explained 9.91% of the variance in the fungal one, while, in spite of insignificant influence, fungi explained 24.93% of the variance in bacterial assemblages.

## DISCUSSION

### Salinization history is a critical factor for *S. europaea* endophytic community structure.

Endophytes can play a major role in plants response to abiotic stresses (e.g., salinity [20]); however, unfavorable environmental factors can affect their diversity and colonization density. In our work, we have observed significant differences in the level of soil salinity between fall and spring at both test sites, but seasons did not affect the distribution of bacterial and fungal endophytes in plant organs. This is contrary to the reports on endophytes of nonhalophytic plants, e.g., Quercus ilex, *Tinospora cordifolia*, *Salix alba*, *S. caprea*, and Betula pendula, where endophytes were investigated (26–28). The reason can be that the interior of the halophyte tissues provides a relatively stable and protective environment for diverse communities of endophytic microbes compared to the saline soil, which is subject to wide fluctuations in the osmotic potential (29).

The diversity of endophytic fungi classified to the genus level in our study was low compared to the diversity seen in endophytic bacterial communities. Previous data revealed that bacterial communities in the rhizosphere exhibit greater richness than endophytes in the organs of halophytes (26, 27). The composition of fungal communities can be affected, as in the case of bacteria, by the soil characteristics. The type of soil acts as a filter for microbial species and, since most endophytes derive from soils, the soil characteristics play an important role (30). While the pH and soil texture are often correlated with the soil bacterial community composition, the fungal community is rather associated with the changes in soil nutrient status (31). Analysis of root endophytic fungal colonization in the halophytes *Salicornia patula* and *Arthrocnemum macrostachyum* revealed that fungal colonization was affected by the differences in soil salinity and host age (32). The relatively low microbial diversity in the shoots of *S. europaea* may be due to the selection imposed by a large salt concentration in this organ.

The differences in bacterial community composition in roots and rhizosphere of *S. europaea* at the test sites (S1 and S2) investigated in this study were also observed by Szymańska et al. using the phospholipid fatty acid analysis method (33). These researchers observed a higher microbial biomass at S1, where the salinity is of natural origin and occurred earlier than at S2.

Because endophytes originate predominantly from the soil, we show that the differences in origin of salinity and ecological characteristics of the test sites may influence endophytic community structure and that soil properties may be an important selection factor shaping their pools (31, 32). Supporting this view, a clear influence of soil properties on the existence of unique endophytes of *S. europaea*, specific to the geographical regions in different countries, such as Japan (34), Slovenia (12), Canada (35), China (25, 36, 37), and Poland (38), was demonstrated. On the other hand, seasons did not influence *S. europaea* endophytic community structure in spite of differences in salinity. This might mean that it was not the salinity itself, but the salinization history, which was causing this variation. Alternatively, the salinity effect might have been so strong that it overrode the influence of seasons.

Differences in the origin of salinity (natural versus anthropogenic) and in consequence the time of microbial community development might be a cause of only *Marinimicrobium* OTUs being detected as a core microbiome in roots of *S. europaea*. Bacteria of the genus *Marinimicrobium* (*Gammaproteobacteria*) are halophilic and/or halotolerant, are strictly aerobic (39), and were never found to live *in planta*. The representatives of this genus were previously isolated from hypersaline surface sediments of the southern arm of Great Salt Lake (Utah) (40), tidal flat sediment of the South Sea in South Korea (the Korea Strait) (39), and the marine solar saltern of the Yellow Sea, South Korea (41). Further work is required to determine the role of *Marinimicrobium* in halophytes.

### Salinity acts as an environmental filter for *S. europaea* bacterial endophytic community both in shoots and roots.

The overall patterns of endophyte abundance, richness, and composition were not only influenced by test sites but also differed between organs. A similar situation was observed in many other plants, e.g., *Tinospora cardifolia* (27). In the present study, we analyzed both the roots and shoots and found higher endophytic community diversity in the former. These results are consistent with those reported by Mora-Ruiz et al. in the halophyte *Arthrocnemum macrostachyum* (42). The greater species richness in roots might be due to their contact with soil, given that most endophytes are derived from soil (43) and migrate from the roots (primary site of interaction between plants and soil) to other tissues (44). Although *Salicornia* is a nonmycorrhizal plant, the abundance and specificity of fungal root endophytes can substitute for the presence of typical mycorrhizae by providing specific functions, e.g., mediating in nutrient transfer from soil into the plant.

Bacterial root endophytes of *S. europaea* were dominated by *Proteobacteria*, specifically class *Gammaproteobacteria*. The greater abundance of *Proteobacteria* identified among halotolerant endophytes compared to rhizospheric soil was also observed in studies of Szymanska et al. involving *S. europaea* (45) and another halophytic plant, *Aster tripolium* L. (33), analyzed using culture-dependent methods. The most abundant phyla of endophytic bacteria observed in our work were also commonly found in other halophytes, e.g., *Halimione portulacoides* or *Salsola imbricate* (46, 47). Thus, we suggest that they may play an important role in the ecology of halophytes. The genus *Kushneria* (family *Halomonadaceae*) was found to be characteristic for the shoots of *S. europaea*. This genus is halophilic and mainly found in saline and hypersaline habitats (48). The bacterial microbiome composition in the rhizosphere of *Salicornia* plants and bulk soils collected by Mapelli et al. (49) from hypersaline ecosystems in Tunisia unveiled the occurrence of the high diversity of *Halomonadaceae* members. That study suggests that *Kushneria* acts as a plant growth promoter that is able to fix atmospheric nitrogen, produce ammonia, display protease activity, and play a role as a biocontrol agent. We found *Saccharospirillum* to be characteristic for the roots of *S. europaea*. This genus belongs to the family *Saccharospirillaceae* and is moderately halophilic, mesophilic, and facultatively alkaliphilic, growing at salinities of 0.5 to 5 % (wt/vol) NaCl, with an optimum at 2 % (50). Moderate halophilic nature may explain its higher abundance in roots than in the salt-accumulating shoots.

The PICRUSt tool was used to evaluate potential functions of the bacterial community, which may enable us to understand how *S. europaea* associated microbiome is able to adapt to high-salt environments and also predict potential ecological roles of the observed organisms. However, the results of such an analysis need to be interpreted with care, since this tool passes on all the biases inherent to amplicon sequencing, as well as depends on a database for unknown organism genome reconstruction. Ten metabolic pathways were differently represented between plant organs. Namely, pathways related to carbohydrate metabolism, e.g., sucrose metabolism, were more common in roots. This might be caused by the greater diversity of complex carbohydrates in roots (51) that would need various enzymes to be utilized by bacteria (52). The complex carbohydrates might be digested to simpler sugars serving as osmolytes and participating in osmotic adjustment (53). Likewise, exposure to frequent salt stress may explain why the bacterial endophytes in this study have an abundance of genes associated with dormancy/sporulation or stress proteins (54). Endophytes escape plant’s defense mechanisms by activating genes involved in glutathione metabolism. Glutathione maintains the proper oxidation state of protein thiols and protects cells from the action of low-pH, chlorine compounds and oxidative as well as osmotic stresses (55). Carotenoid pigments are essential for photosynthetic growth in higher plants and protection against photooxidation (56). The endophytic bacteria may probably contribute by increasing the carotenoid content in the host (57). The simulated bacterial metagenome contained a high number of genes encoding enzymes potentially involved in the detoxification of reactive oxygen species (ROS), particularly in the regulation of redox potential. Plants produce a range of ROS in response to abiotic stress (e.g., salt stress) or colonizing microorganisms that elicit an oxidative burst. Since we cultured many potentially pathogenic fungi from the plant material used in this study (B. U. Furtado, S. Szymańska, and K. Hrynkiewicz, unpublished work), we suggest that mitigation of excess oxidative stress by endophytic bacteria may be beneficial for the host. The greater frequency of oxidative stress response genes in root bacterial communities suggests that the plant response to bacteria is the more important source of stress than salinity, which is higher in shoots (58). However, why the genes involved in the rest of pathways are apparently more common in roots than in shoots still remains to be elucidated.

The root endophytic fungi were dominated by phylum *Ascomycota*, and among them, the family *Pleosporaceae* turned out to be the most abundant in this study. This is consistent with other studies where pleosporalean fungi were found to be the dominant colonizers in halophytes (34) and in plants grown under arid conditions (59). These fungi are common endophytes in both coastal and inland arid soils (60). The references mentioned above suggest an important role of endophytes from the family *Pleosporaceae* in halophytic plants. However, mutualistic interaction between these fungi and plants are not confirmed so far. Among the fungi, *Paradendryphiella arenariae* was the only species present in all sample types in this study but found more frequently in roots. This finding is consistent with earlier reports on this species that was isolated from *S. europaea* roots in Japan (34). *Alternaria chlamydospora* was found in the shoots and roots only at the less-saline site, S2. Muhsin and Booth studied the composition of *Alternaria* assemblages in six different halophytes—*Atriplex patula* L., *Glaux maritima* L., *Hordeum jubatum* L., *Puccinellia nuttalliana*, *S. europaea*, and *Suaeda depressa*—throughout the growing season. Alternaria alternata and *A. chlamydospora* turned out to be specific for *S. europaea* (35).

Notably, some endophytic microorganisms found in our study were only reported from marine environments to date. These are the predominant *Salicornia* endophytic bacteria belonging to *Gammaproteobacteria*, such as *Aestuaricella*, *Marinimicrobium*, *Pseudoalteromonas*, and *Salegentibacter*. We also describe fungal endophytes, such as *Paradendryphiella*, *Neodevriesia*, and *Neocamarosporium*, that were reported in many other halophytic plants (see Table S1A and B in the supplemental material).

### The fungal community may be predicted based on the bacterial one.

Prior studies on the endophytic community in plants focused either on bacteria or fungi. However, as no organism thrives in isolation, the interaction of bacterial and fungal communities in different plant species can be of great relevance. The significant influence of the *Salicornia* bacterial community on the fungal one was found by means of cocorrespondence analysis (CoCA). Bacteria may positively affect fungal activity by producing cellulases and pectinases that increase accessibility of substrates to the fungi (61). In addition, bacteria may also decompose solutes that are toxic to particular fungi (61) and may increase the nitrogen levels available for them (62). Many researchers hypothesized that a mutualistic relationship exists, in which the bacteria utilizing fungal exudates supply nitrogen to fungi (63). Several cases of stimulation of spore germination by spore-associated bacteria have been reported (64, 65). Finally, a multitude of antifungal strategies has been revealed in bacteria, including the production of inhibitory factors such as HCN, antibiotics, lytic enzymes, and volatiles, as well as nutrient-sequestering factors such as iron-chelating siderophores (66–68). Fungi also influence bacteria by facilitating the penetration of bacteria into leaf tissue, where both groups of microorganisms can degrade specific polymers into smaller molecules that are subsequently assimilated (61). Furthermore, fungal hyphae can act as a carrier for bacterial transport, enabling bacteria to colonize new niches faster (69). The exact mechanism and direction of the two communities' influence on one another still remains to be elucidated and would require further studies, such as metatranscriptomic ones.

### Conclusion.

Our results highlight the fact that the history of salinization, and not the salinity itself, is the factor causing differences in community structure between investigated sites. *Saccharospirillum* was characteristic for the roots, and *Kushneria* was found mainly in the shoots, while *Paradendryphiella arenariae* was the most common among fungal endophytes of *S. europaea*. Moreover, the *Salicornia* response to microbes seemed to be a more important source of oxidative stress for bacteria than salinity. Finally, our results showed that the bacterial and fungal communities interact with each other, which led us to speculate that they are codependent, which initiates the need for further research.

## MATERIALS AND METHODS

### Site description and plant sample collection.

Two saline sites were sampled during two seasons (fall 2015 and spring 2016) (see Fig. S1 in the supplemental material). The sites are located in Kujawy region in Central Poland. Site 1 (S1; N52°53′, E18°47′) is located in the vicinity of three brine concentration towers in the Spa Park in Ciechocinek. This natural area favors the salt-marsh vegetation since it is associated with the occurrence of salt springs and saline groundwater in connection with Zechstein rock-salt deposits that are uplifted in the form of salt domes (18). Site 2 (S2; N52°48′, E18°15′) is a meadow located next to waste ponds of the soda factory in Inowroclaw. The long-term deposition of semifluid waste from the factory into sedimentary ponds situated directly on permeable grounds has led to groundwater pollution (19). Three plots (10 × 10 m, biological replicates) were chosen at random at each site. Three blocks of soil (20 × 20 × 20 cm) were randomly sampled from each plot in each of the two time points: fall (F; 21 September 2015) and spring (S; 9 June 2016). The blocks were immediately transferred to the laboratory and processed. The plants were gently uprooted and washed with sterile distilled water and surface sterilized according to Szymańska et al. (45) (Fig. S2). Approximately 500 mg of shoots and roots per each variant of the experiment was weighed and stored in 2-ml vials at –80°C for further processing.

### Physicochemical analysis of soils.

The soil samples were air dried and passed through a 2-mm mesh sieve. The soil was analyzed according to the following methods. The TOC content was determined using a CNS Variomax analyzer, the CaCO_3_ content was determined by the Scheibler volumetric method (70), the electrical conductivity (EC_e_) was determined by conductometry, the pH_e_ was determined potentiometrically, and the saturation percentage (SP) was determined gravimetrically as described by van Reeuwijk (71). The concentrations of ions in the extract were determined as follows: Na^+^, K^+^, Ca^2+^, and Mg^2+^ by the AAS method, Cl^–^ argentometrically, SO_4_^2–^ turbidimetrically, and HCO_3_^–^ acidimetrically (71).

### DNA extraction, library construction, and sequencing.

Portions (500 mg) of frozen plant tissue samples were homogenized with a bead beater (FastPrep-24 MP Biomedicals), and total DNA extraction was performed using a DNeasy plant minikit (Qiagen) according to the producer’s protocol. The DNA was quantified fluorometrically (Qubit 2.0), and the quality was assessed spectrophotometrically (NanoDrop 2000).

A two-step PCR (72) was carried out in order to generate bacterial 16S rRNA and fungal ITS amplicon libraries. Amplification, purification, quantification, and sequencing were performed as described earlier by Thiem et al. (73). Briefly, in the first-PCR-round V3-V4 fragments of bacterial 16S rRNA genes and ITS regions of eukaryotic ribosomal DNA (rDNA) were amplified with the 357f/786r primer pair (72), and ITS1 and ITS2 (74) were amplified with M13/M13R overhangs using metagenomic DNA as the template. In the second round, the amplicons were converted to the Illumina sequencing library by PCR with M13/M13R primers bearing barcodes and P5/P7 adapters. Libraries were purified twice with SPRI beads (GE Healthcare), quantified using the KAPA library quantification kit for Illumina (Roche), and sequenced using MiSeq (Illumina) with custom sequencing primers being high-pressure liquid chromatography-purified versions of the first-round primers for R1 and R2 and reverse complement of the reverse first-round primer for the index read.

### Bioinformatics and statistical analysis.

Raw reads were denoised and merged using the DADA2 R package to yield amplicon sequence variants (ASVs) and information on their abundance (75). Then, the denoised sequences were separated into bacterial and nonbacterial (ITS) sequences via classification with the naive Bayesian classifier implemented in mothur (76) using the SILVA v.123 database (77, 78). Sequences that were unclassified were regarded as fungal and then processed separately.

Bacterial sequences were processed as described earlier by Gołębiewski et al. (72), while fungal sequences were processed with ITSx (79), and all fungal ITS1 sequences were used in the downstream analysis. The 16S rRNA ASVs processing pipeline was implemented in mothur v.1.39 and consisted of alignment against SILVA v.123, screening for sequences covering the desired region of the alignment (from positions 6428 to 22440), filtering all-gap- and terminal gap-containing columns from the alignment, and the removal of residual noise via preclustering and chimera identification using UCHIME. The final set of sequences was then clustered to 0.03 dissimilarity OTUs with the Opti-MCC algorithm (80), and an OTU table was constructed. The denoised fungal sequences (ASVs) were dereplicated, and OTUs were constructed using vsearch (81) at a 0.03 dissimilarity level. Singletons, as well as doubletons (OTUs consisting of one or two sequences only), were removed from both data sets. The sequences were classified with a naive Bayesian classifier (82) using SILVA v.123 (bacteria) and ITS1 extracted from UNITE v.7 (fungi), and the nonbacterial and nonfungal sequences were removed from the respective sets. The final data were subsampled to 500 (bacteria) and 300 (fungi) sequences per sample 20 times; OTUs were constructed as described earlier, and the resulting OTU tables were averaged over the 20 subsamples.

The core microbiome was delineated by using the get.coremicrobiome function of mothur with an abundance threshold of 0.5%. The lower threshold was used because no core OTUs were identified at the standard 1% threshold (83).

Statistical analyses were performed using the vegan package v.2.4-5 (39) in R (v3.4.4). The observed OTUs, Shannon’s diversity (H′), and Chao1 indices were calculated using the diversity and estimateR functions of the vegan package, while Shannon’s evenness was computed by dividing the H′ by the logarithm of the number of OTUs. NMDS analysis was performed with meta-MDS using Bray-Curtis and Morisita-Horn distances calculated from Wisconsin-normalized data. Permutational analysis of variance (PERMANOVA; adonis function) was performed to test whether endophytic bacterial and fungal communities were significantly different among tissues or sample sites, and PERMDISP (betadisper) was used to check for homogeneity of variance. Canonical correlation analysis (CCA) was used for community-constrained ordination, and soil parameters were selected as constraints in CCA. Influence of communities on one another was assessed using CoCA (84) implemented in the cocorresp R package (85). A total of 999 permutations were used in permutational tests.

Prediction of putative function sets was performed with PICRUSt as described earlier in Thiem et al. (73).

### Data availability.

The bacterial and fungal sequence data generated in this study using MiSeq have been deposited and are available in the NCBI Sequence Read Archive (SRA) under BioProject ID PRJNA412808.

## Supplementary Material

Supplemental file 1
