# Supplementary material for: Bacterial and Fungal Endophytic Microbiomes of Salicornia europaea
Source: Appl Environ Microbiol. 2019 Jun 17;85(13):e00305-19. doi: 10.1128/AEM.00305-19 (PMC6581177; doi:10.1128/AEM.00305-19)
Supplement: Supplemental file 1 [file AEM.00305-19-s0001.pdf]

Exploring bacterial and fungal endophytic microbiomes of *Salicornia europaea*

Bliss Furtado[1,3], Marcin Gołębiewski[2,3], Monika Skorupa[3], Piotr Hulisz[4], Katarzyna Hrynkiewicz[1,3*]

[1]Department of Microbiology, Faculty of Biology and Environmental Protection, Nicolaus Copernicus University, Lwowska 1, 87-100 Torun, Poland

[2]Department of Plant Physiology and Biotechnology, Nicolaus Copernicus University, Lwowska 1, 87-100 Torun, Poland

[3]Interdisciplinary Center for Modern Technologies, Nicolaus Copernicus University, Wilenska 4, 87-100 Torun, Poland

[4]Department of Soil Science and Landscape Management, Faculty of Earth Sciences, Nicolaus Copernicus University, Lwowska 1, 87-100Toruń, Poland

* Corresponding author:

K. Hrynkiewicz

Phone: (+48)-56-6114447; Fax: (+48)-56-6114772

E-mail:hrynk@umk.pl

Table S1 Potential role of *Salicornia* bacterial and fungal endophytic strains reported in this study.

A

Bacterial genus	NCBI accession no.	Group	Source of isolation	Potential function	References
Aestuariicella	KF982858	ND	sea tidal flat	hydrocarbon-degrading	Naysim et al. 2015
Arcobacter	JF521610	endophyte	Bryopsis specimens	ND	Hollants et al. 2011
Flavimarina	KX664463	endophyte	*Salicornia herbacea*	hydrolyse gelatin	Cho et al. 2017
Halomonas	LWGO00000000	endophyte	roots of *Cyperus conglomeratus*	PGP	Lafi et al. 2016
Idiomarina	KP706811	ND	coastal plain	ND	You et al. 2015
Kushneria	LN651144	endophyte	leaves of Salicornioideae	ND	Mora-Ruiz et al. 2015
Leeuwenhoekiella	DQ294290 and DQ294291	ND	seawater	degradation of organic matter during and following algal blooms	Pinhassi et al. 2006
Lewinella	KX499306	epiphytes	*Caulerpa cylindracea*	ND	Stabili et al. 2017
Marinimicrobium	HQ882175	ND	marine solar saltern	inulinase activity	Li et al. 2012
Marinobacter	PRJEB8726	endophyte / rhizosphere	roots of *Suaeda salsa*	PGP	Yuan et al. 2016
Marivirga	JX500546	ND	rhizosphere	ND	Haldar et al.2015
Massilia	ND	endophyte	stalk of maize plant	IAA producer	Celador-Lera et al. 2016
Nocardioides	AY148081	endophyte	wheat roots	induce defence	Conn et al. 2008
Planomicrobium	KP209393	endophyte	*Lavandula angustifolia*	antimicrobial activity	Beiranvand et al. 2017
Pseudoalteromonas	AOPM00000000	ND	saltern	enzyme activity	Haba et al. 2013
Psychrobacter	AJ748266	endophyte	*Arthrocnemum macrostachyum*	ND	Mora-Ruiz et al. 2016
Saccharospirillum	KY310592	endophyte	*Halimione portulacoides*	ND	Fidalgo et al. 2017

Salegentibacter	ND	ND	water and sediment samples	cytotoxic effects in mammals	Díaz-Cárdenas et al. 2017
Salinicola	KT036407 S	endophyte	*Spartina maritima*	PGP	Mesa et al. 2015
Thalassospira	KU587165	endophyte	roots of Caliph medic (*Medicago truncatula*)	ND	Yaish et al. 2016
Vibrio	KT036401	endophyte	*Spartina maritima*	PGP	Mesa et al. 2015

B

**Fungal genus**	**NCBI accession no.**	**Group**	**Source of isolation**	**Potential function**	**References**
Plenodomus	KY064055	pathogen	Chrysanthemum sp.	ND	Marin-Felix et al. 2017
Paradendryphiella	KT207744	ND	Marine brown algae, Sargassum sp.	antifungal and antioxidant activity	Hong et al. 2015
	AB975313	endophyte	*Salicornia europaea*	ND	Okane et al. 2015
Neodevriesia	NR_132905	ND	leaves of *Afrocarpus falcatus*	saprobic or plant pathogenic	Crous et al. 2014
Neocamarosporium	KX817217	ND	saline lakes of Iran	ND	Papizadeh et al. 2017
Didymella	AB975296	endophyte	*Salicornia europaea*	ND	Okane et al. 2015
Alternaria	EU143251	endophyte	leaves of *Polygonum senegalense*	production of natural cytotoxic metabolites	Aly et al. 2008
	FJ465171	endophyte	leaves of *Sonneratia alba*	secondary metabolites	Kjer et al. 2009

Abbreviations: **PGP-** plant growth promoting, **ND-** not defined

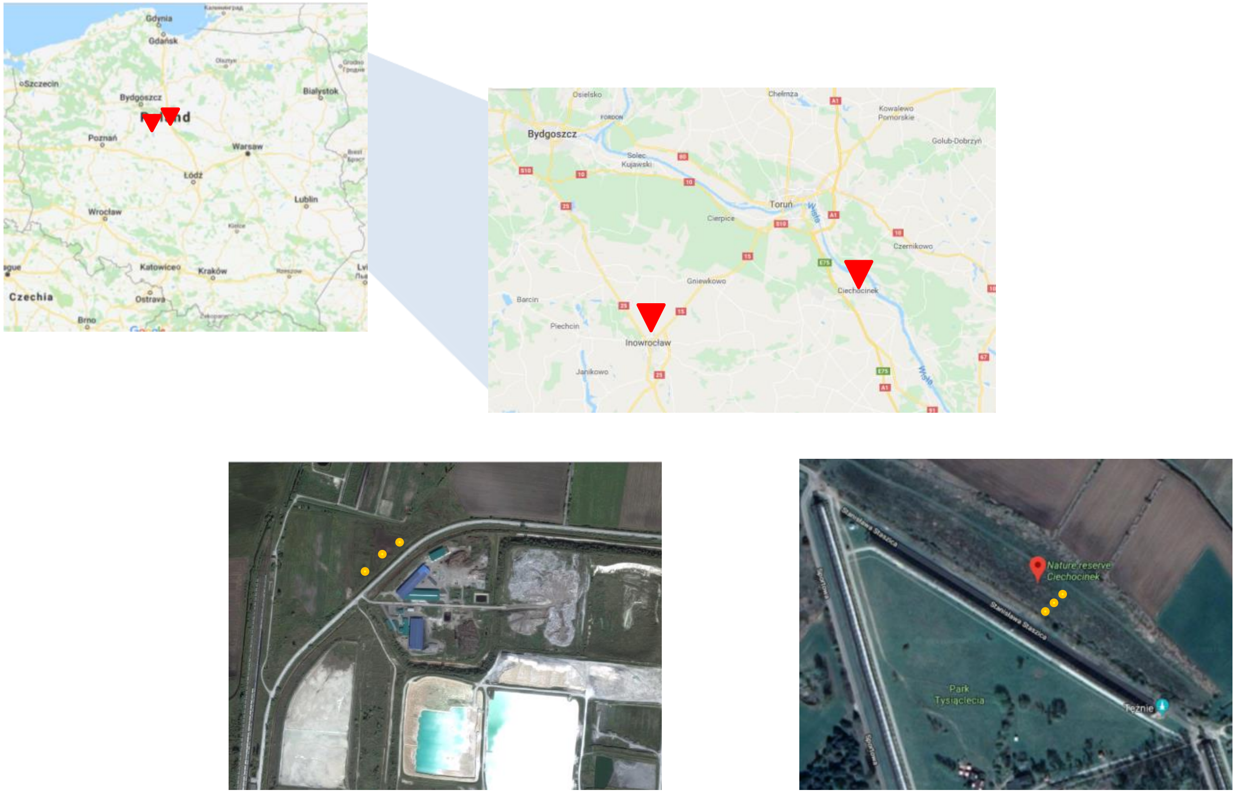

Inowroclaw (S2) **Ciechocinek (S1)**

Fig. S1 Two sample collection sites in Central Poland: The map highlights the two salt affected sites S1- Ciechocinek and S2- Inowroclaw and the three plots of which the three plant biological replicates were collected.

Separate the plant material from soil in a tray (keep soil samples aside for further tests)

⬇

Wash the plant with sterile 2% NaCl (wash 6X) to remove soil debris

⬇

Place the plant on filter paper and blot dry

⬇

Using sterile scalpel cut the roots and shoots for disinfection steps

⬇

Wash roots with 5% H_2O_2 and shoots with 2% H_2O_2 for 5 minutes

⬇

Rinse the root and shoot in sterile 2% NaCl separately (repeat this step 3X)

⬇

Blot dry the disinfected plant tissue using sterile filter papers

⬇

Weigh 500 mg of sample in 2 ml vials

⬇

Store samples in -80^{0}C for DNA extraction

Fig. S2 Summary of plant surface sterilization technique.

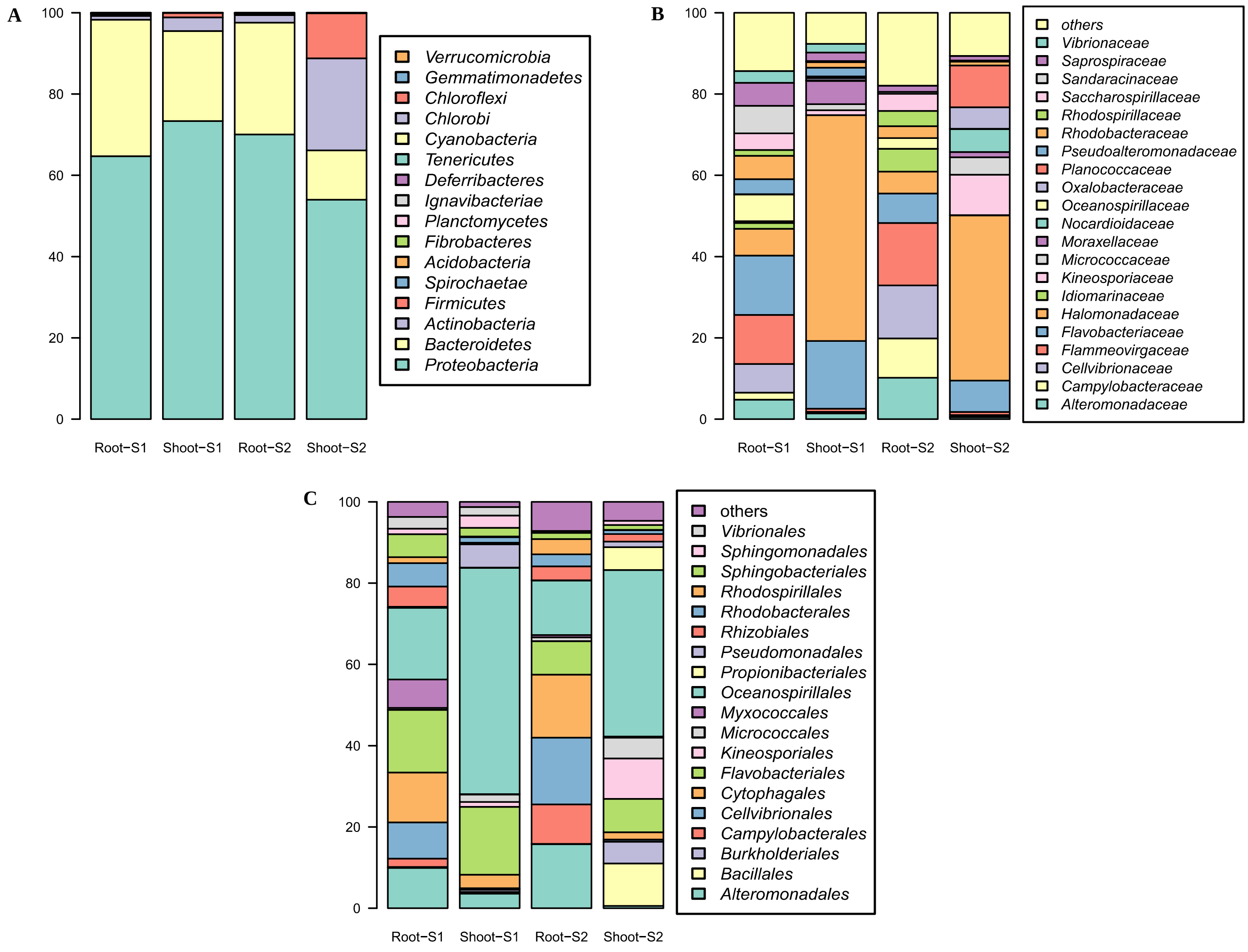

Fig. S3 Endophytic bacterial community structure at the (A) phylum (B) family and (C) order level between test sites and plant organ.

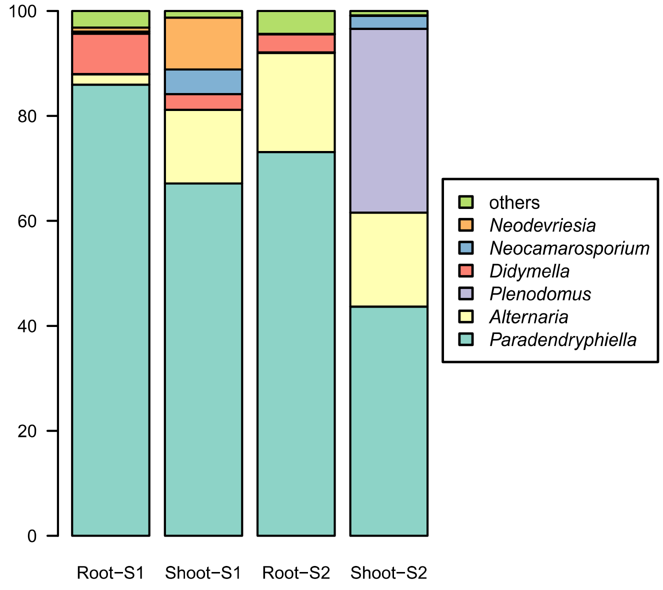

Fig. S4 Endophytic fungal community structure at the genus level between test sites and plant organ.

References

1. Aly AH, Edrada-Ebel R, Indriani ID, Wray V, Müller WEG, Totzke F, et al. Cytotoxic Metabolites from the Fungal Endophyte Alternaria sp. and Their Subsequent Detection in Its Host Plant Polygonum senegalense. J Nat Prod. 2008; 71:972–80. doi:10.1021/np070447m.
2. Beiranvand M, Amin M, Hashemi-Shahraki A, Romani B, Yaghoubi S, Sadeghi P. Antimicrobial activity of endophytic bacterial populations isolated from medical plants of Iran. Iranian Journal of Microbiology. 2017;9(1):11-18.
3. Celador-Lera L., Menéndez E., Flores-Félix J.D., Mateos P.F., Rivas R. Analysis of the PGPB Potential of Bacterial Endophytes Associated with Maize. In: González-Andrés F., James E. (eds) Biological Nitrogen Fixation and Beneficial Plant-Microbe Interaction. Springer, Cham 2016 doi:https://doi.org/10.1007/978-3-319-32528-6_3
4. Cho E-S, Cha I-T, Park J-M, Choi H-J, Lee JH, Roh SW, et al. Flavimarina flava sp. nov., isolated from Salicornia herbacea. Int J Syst Evol Microbiol. 2017;67:4240–5. doi:10.1099/ijsem.0.002292.
5. Conn V M; Walker A R; Franco C M M. Endophytic actinobacteria induce defense pathways in Arabidopsis thaliana. Molecular Plant-Microbe Interactions 2008, 21, 208-218.
6. Crous PW, Shivas RG, Quaedvlieg W, et al. Fungal Planet description sheets: 214–280. Persoonia : Molecular Phylogeny and Evolution of Fungi. 2014; 32:184-306. doi:10.3767/003158514X682395.
7. de la Haba RR, Sanchez-Porro C, Leon MJ, Papke RT, Ventosa A. Draft genome sequence of the moderately halophilic bacterium Pseudo alteromonas ruthenica Strain CP76. Genome Announc. 2013;1. doi:10.1128/genomeA.00268-13.
8. Díaz-Cárdenas, Carolina, Cantillo, Angela, Rojas, Laura Yinneth Sandoval, Tito, Fiorentino, Susana, Robles, Jorge, Ramos, Freddy A., Zambrano, María Mercedes, Baena, Sandra. Microbial diversity of saline environments: searching for cytotoxic activities. AMB Express 2017; 7:223 https://doi.org/10.1186/s13568-017-0527-6
9. Fidalgo C, Rocha J, Proença DN, Morais PV, Alves A, Henriques I. Saccharospirillum correiae sp. nov., an endophytic bacterium isolated from the halophyte Halimione

portulacoides. Int J Syst Evol Microbiol. 2017; 67:2026–30. doi:10.1099/ijsem.0.001914.

10. Haldar, S. and Sengupta, S. Impact of plant development on the rhizobacterial population of Arachis hypogaea: a multifactorial analysis. J. Basic Microbiol., 2015; 55: 922-928. doi:10.1002/jobm.201400683

11. Hollants, J., Leroux, O., Leliaert, F., Decleyre, H., De-Clerck, O., and Willems, A. Who is in there? exploration of endophytic bacteria within the siphonous green seaweed Bryopsis (Bryopsidales, Chlorophyta). PLoS ONE 2011; 6:e26458. doi: 10.1371/journal.pone.0026458

12. Hong, J.-H.; Jang, S.; Heo, Y.M.; Min, M.; Lee, H.; Lee, Y.M.; Lee, H.; Kim, J.-J. Investigation of marine-derived fungal diversity and their exploitable biological activities. Mar. Drugs 2015, 13, 4137–4155.

13. Kjer J, Wray V, Edrada-Ebel R, Ebel R, Pretsch A, Lin W, et al. Xanalteric Acids I and II and related phenolic compounds from an endophytic Alternaria sp. Isolated from the Mangrove Plant Sonneratia alba. J Nat Prod. 2009; 72:2053–7. Doi: 10.1021/np900417g.

14. Lafi FF, Ramirez-Prado JS, Alam I, Bajic VB, Hirt H, Saad MM. Draft genome sequence of Halomonas elongata strain K4, an endophytic growth-promoting bacterium enhancing salinity tolerance in planta. Genome Announc 2016 4(6):e01214-16. doi:10.1128/genomeA.01214-16.

15. Li, AX., Guo, LZ. & Lu, WD. Alkaline inulinase production by a newly isolated bacterium Marinimicrobium sp. LS–A18 and inulin hydrolysis by the enzyme World J Microbiol Biotechnol 2012; 28: 81. https://doi.org/10.1007/s11274-011-0794-3

16. Marin-Felix Y, Groenewald JZ, Cai L, et al. Genera of phytopathogenic fungi: GOPHY 1. Studies in Mycology 2017; 86: 99–216

17. Mesa J, Mateos-Naranjo E, Caviedes MA, Redondo-Gómez S, Pajuelo E and Rodríguez-Llorente ID Endophytic cultivable bacteria of the metal bioaccumulator Spartina maritima improve plant growth but not metal uptake in polluted marshes soils. Front. Microbiol. 2015; 6:1450. doi: 10.3389/fmicb.2015.01450

18. Mora-Ruiz, M. D. R., Font-Verdera, F., Orfila, A., Rita, J., and Rossel-Mora, R. Endophytic microbial diversity of the halophyte Arthrocnemum macrostachyum across

plant compartments. FEMS Microbiol. Ecol. 2016; 92, 1–10. doi:10.1093/femsec/fiw145

19. Mora-Ruiz, M. R., Font-Verdera, F., Díaz-Gil, C., Urdiain, M., Rodríguez-Valdecantos, G., González, B., et al. Moderate halophilic bacteria colonizing the phylloplane of halophytes of the subfamily Salicornioideae (Amaranthaceae). Syst. Appl.Microbiol. 2015; 38, 406–416. doi: 10.1016/j.syapm.2015. 05.004

20. Naysim Lo, Kyung Hyun Kim, KyunghwaBaek, Baolei Jia and Che Ok Jeon Aestuariicella hydrocarbonica gen. nov., sp. nov., an aliphatic hydrocarbon-degrading bacterium isolated from a sea tidal flat. International Journal of Systematic and Evolutionary Microbiology 2015, 65, 1935–1940 DOI 10.1099/ijs.0.000199

21. Okane, I., and Nakagiri, A. Assemblages of endophytic fungi on Salicornia europaea disjunctively distributed in Japan: towards clarification of the ubiquity of fungal endophytes on halophytes and their ecological roles. Curr. Sci. 2015; 109, 62–71

22. Papizadeh, M., Wijayawardene, N.N., Amoozegar, M.A. et al. Neocamarosporium jorjanensis, N. persepolisi, and N. solicola spp. nov. (Neocamarosporiaceae, Pleosporales) isolated from saline lakes of Iran indicate the possible halotolerant nature for the genus Mycol Progress 2018 17: 661. https://doi.org/10.1007/s11557-017-1341-x

23. Pinhassi J, Bowman J, Nedashkovskaya O, Lekunberri I, Gomez-Consarnau L, Pedrós-Alió C. Leeuwenhoekiella blandensis sp. nov., a genome-sequenced marine member of the family Flavobacteriaceae. Int J Syst Evol Microbiol 2006; 56(7):1489-1493 doi:10.1099/ijs.0.64232-0

24. Stabili Loredana, Rizzo Lucia, Pizzolante Graziano, Alifano Pietro, Fraschetti Simonetta. Spatial distribution of the culturable bacterial community associated with the invasive alga Caulerpa cylindracea in the Mediterranean Sea, Marine Environmental Research, 2017; Vol. 125, Pg. 90-98, ISSN 0141-1136, https://doi.org/10.1016/j.marenvres.2017.02.001.

25. Yaish MW, Al-Lawati A, Jana GA, Vishwas Patankar H, Glick BR. Impact of soil salinity on the structure of the bacterial endophytic community identified from the roots of Caliph medic (Medicago truncatula). PLoS One. 2016; 11:e0159007. doi:10.1371/journal.pone.0159007.

26. You Y-H, Jin YJ, Kang S-M, Oh S, Lee M-C, Kim J-G. Plant growth-promoting activity and identification of endophytic fungi isolated from native plant in East coast. Korean J Microbiol. 2015; 51:14–20. doi:10.7845/kjm.2015.5005.
27. Yuan, Z. et al. Specialized microbiome of a halophyte and its role in helping non-host plants to withstand salinity. Sci. Rep. 2016; 6, 32467; doi: 10.1038/srep3.
